# Achieving reliable patient reported outcomes collection to measure health care improvement in a learning health network: lessons from pediatric rheumatology care and outcomes improvement network

**DOI:** 10.3389/fped.2024.1443426

**Published:** 2025-01-08

**Authors:** Nancy Pan, Esi M. Morgan, Meghan Ryan, Beth Gottlieb, Julia G. Harris, Tzielan Lee, Y. Ingrid Goh

**Affiliations:** ^1^ Division of Pediatric Rheumatology, Hospital for Special Surgery, New York, NY, United States; ^2^ Department of Pediatrics, Weill Medical College of Cornell University, New York, NY, United States; ^3^ Department of Pediatrics, Seattle Children's Hospital & University of Washington School of Medicine, Seattle, WA, United States; ^4^ Department of Pediatrics, UnityPoint Health-Blank Children’s Hospital, Des Moines, IA, United States; ^5^ Long Island Jewish Medical Center, Northwell Health, New Hyde Park, NY, United States; ^6^ Department of Pediatric Rheumatology, Cohen Children’s Medical Center, Queens, NY, United States; ^7^ Division of Rheumatology, Department of Pediatrics, Children’s Mercy Kansas City, Kansas City, MO, United States; ^8^ University of Missouri-Kansas City School of Medicine, Kansas City, MO, United States; ^9^ Division of Pediatric Rheumatology, Department of Pediatrics, Stanford Medicine Children’s Health, Stanford, CA, United States; ^10^ Stanford University School of Medicine, Stanford, CA, United States; ^11^ Division of Rheumatology, The Hospital for Sick Children, Toronto, ON, Canada; ^12^ Child Health Evaluative Sciences, SickKids Research Institute, Toronto, ON, Canada

**Keywords:** patient reported outcomes, pediatric rheumatology, quality of life, outcome measures, juvenile idiopathic arthritis

## Abstract

**Introduction:**

Data from the Pediatric Rheumatology Care and Outcomes Improvement Network (PR-COIN) registry suggests that reliable collection of patient-reported outcomes (PROs) varies across sites. The objective of this study was to better understand the practices of collecting PROs at PR-COIN sites.

**Methods:**

A REDCap survey was sent to the lead representative for each PR-COIN site. Registry data were analyzed to better understand the completion rates of PROs. Interviews of physician leaders of high performing sites were conducted by videoconference, audiotranscribed and themes were summarized. Quantitative data were analyzed using descriptive statistics and qualitative data were thematically analyzed.

**Results:**

All 23 PR-COIN sites responded to the survey. PROs were collected by 21/23 (91%) sites. Arthritis-related pain intensity, morning stiffness, and physical function were the top three collected PROs ( Supplementary 3 and 4). PROs were collected using paper, electronically or in combination, with most sites collecting PROs only on paper. PROs were manually scored at most sites. Among sites with electronic PRO collection, 42% did not have automatic transfer of scores into the electronic medical record. Facilitators to successful collection of PROs included availability of staff, training, and culture. Barriers to PRO collection cited were limited time, lack of infrastructure, and lack of staff. Completion rates of PROs in the registry in top 4 performing centers for morning stiffness was 100%, overall well-being and pain intensity scores ranged from 93%–98%, and for physical function 69%–94%. Interviews with physician leaders indicated that their site overcame barriers through: integration of PRO collection into workflow, gaining buy-in of stakeholders (clinicians and patients), and automating PRO collection. Interviewees endorsed automation of data collection (e.g., self-completion on tablets) and automated transfer to electronic medical record (EMR) as key components enabling reliable PRO collection.

**Conclusions:**

Through understanding our current ability to systematically collect PROs across all sites in PR-COIN and exploring successful implementation of PRO collection both within and outside our learning health network, we share lessons learned and identify the most influential factors for successful PRO collection in pediatric rheumatology.

## Introduction

As healthcare moves towards more patient-centered care, it is imperative to examine methods of integrating patients’ opinions into clinical assessments and decision-making. In pediatrics this is achieved by obtaining input from either the patient themselves or, in cases where the patient does not have the developmental capacity, their proxy. Patient-reported outcomes (PROs), defined as “any report of the status of a patient's health condition that comes directly from the patient without interpretation of the patient's response by a clinician or anyone else”, are an important tool for measuring patient outcomes (1). Patient-reported outcome measures (PROMs), validated questionnaires which are used to measure PROs, are completed by patients and their proxies to inform their healthcare teams about their perception of their health status and quality of life.

Pediatric Rheumatology Care and Outcomes Improvement Network (PR-COIN) (2) is a learning health network (LHN) dedicated to improving healthcare delivery and patient outcomes through quality improvement methodology (3). Patient data across the network are collected in a central registry (4). Patient engagement is a central component of a LHN, and the patient voice is integral to care through shared decision-making. The PR-COIN LHN's focuses on outcomes improvement prioritizes disease control, relief of pain, and optimization of physical function through a “treat to target” strategy (5). Striving for complete data collection is a critical first step toward understanding disease activity status, gaps in care, and ultimately, planning impactful interventions to improve health outcomes. As this LHN is a collaborative between patients and families, the collection is PROs is important for patient-reported outcome data in the case of a LHN that is co-produced with patients and families and prioritizes outcomes that are important to patients. Qualitative research with patients and families indicate that pain, physical function and patient perception of overall well-being are outcomes they prioritize to be measured in longitudinal observational studies and clinical trials (6) and are therefore collected as quality measures in PR-COIN (3).

Despite the recognition of the importance of these health domains to patients and intent for reliable collection of these measures, collection of PROs within PR-COIN varies across sites. To better comprehend the various practices for collecting PROs within PR-COIN, the PRO Standardization Workgroup conducted a survey of sites to determine which PROs were being collected, to understand operational processes to PRO completion, and to identify facilitators and barriers to collecting PROs. The goals of this paper are to: (1) report the results of this survey, (2) present current performance on PRO data reporting in the PR-COIN registry and (3) present results of interviews highlighting sites that successfully implement systems to collect and transfer completed PRO data to the electronic medical records (EMR) and registry.

## Materials and methods

The PR-COIN registry was approved by Seattle Children's Institutional Review Board (IRB), which serves as the IRB of record for Seattle Children's Hospital.

A REDCap survey was sent to the lead representative for each PR-COIN site. Lead representatives were asked to consult with their site members prior to completing the survey. Survey questions included how PROs were collected, which PROs were collected, facilitators and barriers to collection (Supplementary 1). Quantitative data were analyzed using descriptive statistics and qualitative data were thematically analyzed.

Registry data was analyzed to better understand the completion rates of patient reported data such as morning stiffness, pain intensity scores, physical function, and overall well-being.

The interviews with site physician leaders were deemed exempt by Hospital for Special Surgery's Institutional Review Board.

PR-COIN conducts biannual meetings where sites within the LHN share experiences to facilitate learning. Four physician leaders who previously reported successful implementation of PRO collection were invited to participate in a one-time virtual interview (with NP) where they shared how PROs were collected at their site, identified key resources that facilitated documentation of PROs in their EMR, barriers they had to overcome, and share best practices (Supplementary 2). A summary of the interview was provided to each participant for review and approval.

One-on-one interviews of physician leaders were conducted by videoconference by NP and audiotranscribed. Preliminary thematic analysis was conducted independently by NP and EMM, and agreement on major themes achieved through discussion. Subsequently, two separate reviewers (IG and SJ) identified themes using inductive thematic analysis utilizing NVivo 15 software by Lumivero (7).

## Results

All 23 PR-COIN sites responded to the survey. PROs are collected by 21/23 (91%) sites which variably measured patients’ perception of their condition or symptoms of their condition, self-management, medication side effects, ability to do activities of daily living, and mental health status (Table 1). PROs were collected for both clinical and/or research purposes. The top three collected PROs for both clinical and research purpose were arthritis-related pain intensity score, morning stiffness and physical function as measured by the Childhood Health Assessment Questionnaire (CHAQ) score (8). The PR-COIN registry collects morning stiffness using delineated increments of time and the survey responses reflect the number of sites reporting morning stiffness in the registry. Five of 23 (21.7%) sites indicated that their institution had mandated certain questionnaires be collected throughout their institution e.g., assessments of mental health and suicide screening.

**Table 1:** Patient reported outcome measure collection in PR-COIN.

Patient reported outcome measure		
Pain-intensity score	19/23	82.6%
Morning stiffness	18/23	78.3%
Child health assessment questionnaire (CHAQ)/Health assessment questionnaire (HAQ)	17/23	73.9%
Patient global-overall well-being	14/23	60.9%
Patient global assessment	13/23	56.5%
Review of systems	13/23	56.5%
PROMIS-Pain interference	6/23	26.1%
Transition readiness	6/23	26.1%
Patient health questionnaire-9 (PHQ-9)	5/23	21.7%
PROMIS-upper extremity	5/23	21.7%
Patient global-disease activity	4/23	17.4%
PedsQL RHE child	4/23	17.4%
PedsQL RHE parent	4/23	17.4%
PROMIS-mobility	4/23	17.4%
PedsQL core child	3/23	13.0%
PedsQL core parent	3/23	13.0%
Bath ankylosing spondylitis disease activity index (BASDAI)	2/23	8.7%
Patient health questionnaire-2 (PHQ-2)	2/23	8.7%
PROMIS-depressive symptoms	2/23	8.7%
Juvenile arthritis functional assessment report (JAFAR)	1/23	4.3%
Methotrexate intolerance severity score (MISS)	1/23	4.3%
PROMIS-anxiety	1/23	4.3%
PROMIS-fatigue	1/23	4.3%
Quality of my life (QoML)	1/23	4.3%
EQ-5D	0/23	0.0%
Juvenile arthritis functional status index (JASI)	0/23	0.0%
Juvenile arthritis functionality scale (JAFS)	0/23	0.0%
Juvenile arthritis multidimensional assessment report (JAMAR)	0/23	0.0%
Juvenile arthritis quality of life questionnaire (JAQQ)	0/23	0.0%
Outcome measure child health questionnaire (CHQ)	0/23	0.0%
Pain symptom assessment tool (PSAT)	0/23	0.0%

Interestingly, the patient global assessment of overall well-being was collected more often for research purposes rather than clinical purposes. The patient global assessment score was collected on varying scales (0–10 vs. 0–100 range) and varying increments (1 vs. 0.5 vs. 0.1 unit). Nine of 23 (39%) sites indicated that they planned to add additional questions or questionnaires to measure PROs, or PROMs such as PROMIS short form measures (9) in the future.

**Figure 1:**
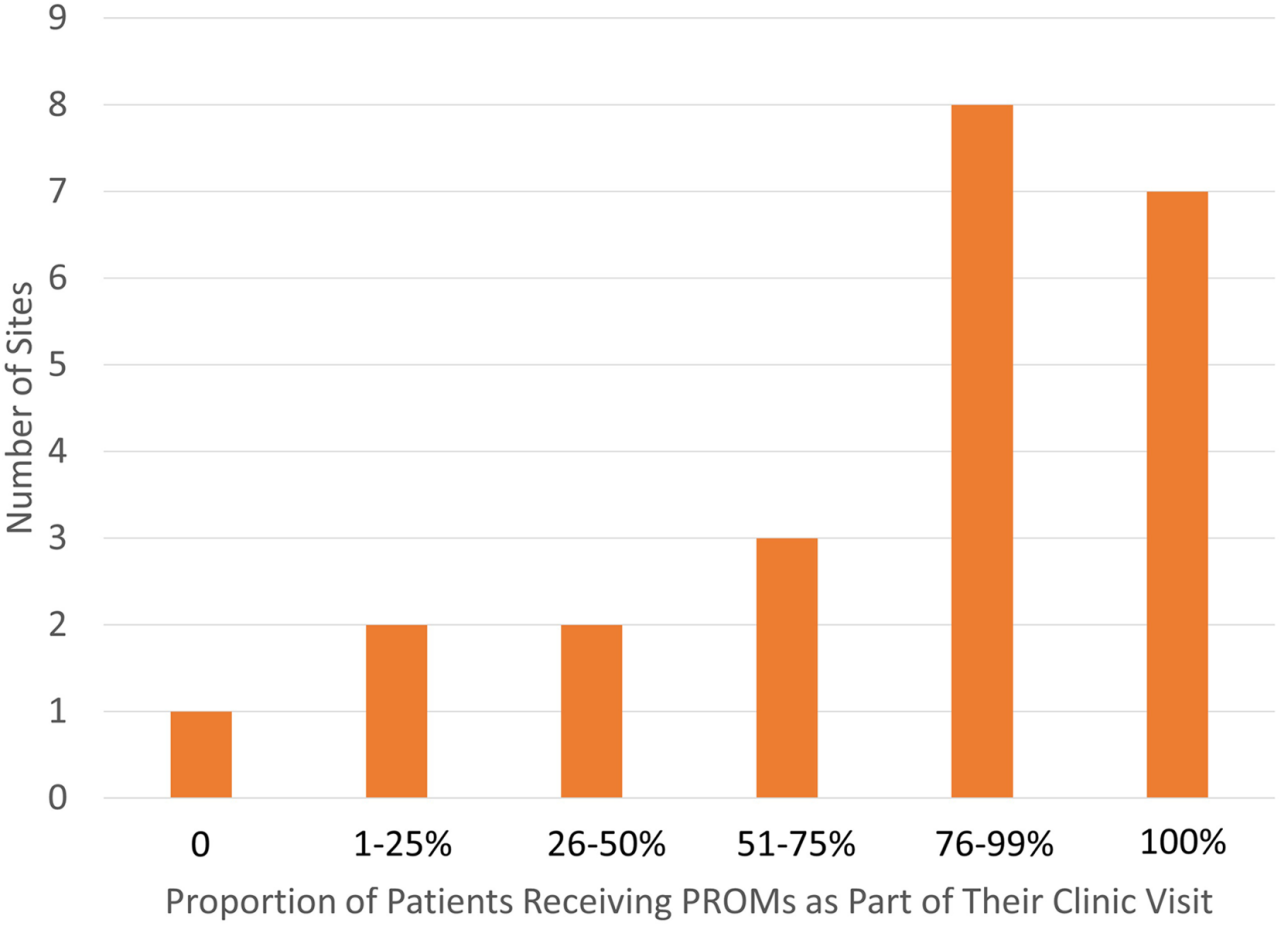
Patients receiving PROMs at PR-COIN sites.

**Figure 2:**
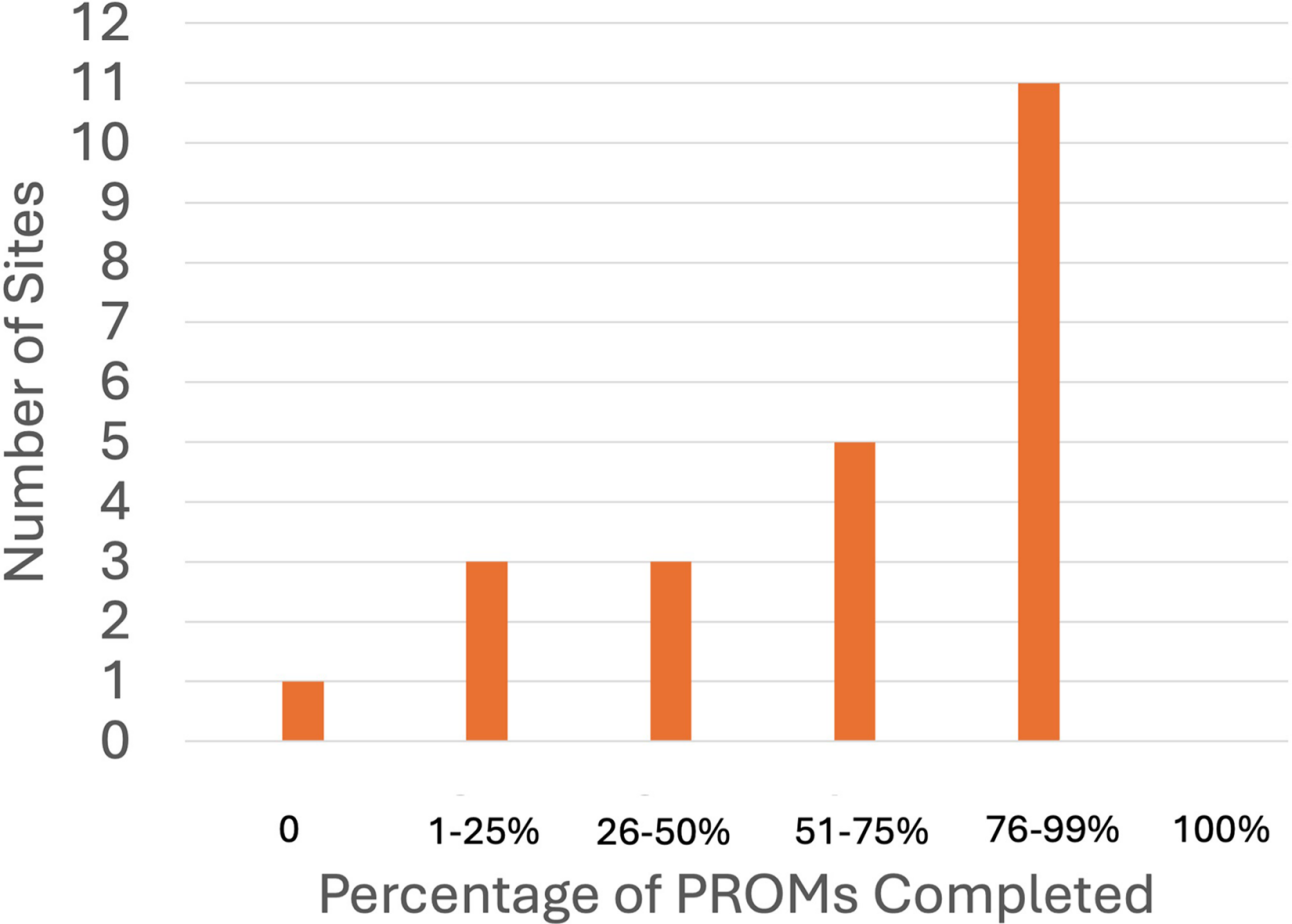
PROMs completion at PR-COIN sites.

One site reported not distributing questionnaires to their patients to complete, whereas seven sites indicated that 100% of their patients received PROMs (Figure 1). Eleven sites reported high reliability of completion of the distributed PROMs, with a 76%–99% completion rate (Figure 2).

Respondents reported that PROMs were collected using both paper and/or electronic methods, with many sites 11/23 (47.8%) collecting PROMs only on paper, fewer 7/23 (30.4%) collected PROMs only digitally, and 5/23 (21.7%) collected PROMs both on paper and digitally. When collected digitally, sites indicated that PROMs were administered using a variety of methodologies: a tablet with data flowing directly into the EMR or REDCap, via patient portal into the EMR, and website.

All sites used EMR systems to document their patient encounters 17/23 (73.9%) Epic (10) 3/23 (13%) Cerner (11), and AllScripts (12) 3/23 (13%).

Sites reported involvement of a variety of individuals in the administration of PROMs, including physicians, nurses, medical assistants, front desk staff, research team members, volunteers, and self-administration by patients/proxies. More than 75% of respondents reported patients had the ability to self-administer PROMs via a tablet in clinic or patient portal survey. PROs were usually completed by either patient or proxy before or during the appointment. Most sites reported that PROMs were completed by more than half of the patients (Figure 1).

Respondents estimated that staff spent three minutes administering PROMs compared to six minutes for patients and proxies to self-administer.

The majority of sites (14/23, 60.9%) reported that PROM scores were manually calculated. Five of 23 sites (21.7%) indicated that the score was electronically calculated, whereas 4 of 23 (17.4%) used either electronic or manual methods to calculate the score. In cases where the scores were manually calculated, individuals performing the calculations were: physician, practitioner, nurse, trainees, medical assistant, research team member, and volunteer.

Respondents estimated that clinical or research staff took about four minutes to manually enter scores into the EMR. Although some sites reported their patients and proxies could complete their PROMs electronically, 5/12 (42%) sites reported that scores could not automatically be imported into the patient's EMR.

Respondents indicated that availability of personnel, training and culture were the greatest facilitators, whereas limited time and lack of staff were the greatest barriers to PROM completion.

Self-reported completion of PROs in this survey cannot be extrapolated to completeness of data entry in the PR-COIN Registry. Data from the PR-COIN registry showed that across the 14 centers submitting PROs, morning stiffness was collected at all sites. Arthritis-related pain scores were collected from 13 of 14 sites (92%) and patient global assessment of overall well-being scores from 13/14 sites (92%). Measures of physical function included the CHAQ, Patient-Reported Outcomes Measurement Information System (PROMIS) mobility and PROMIS upper extremity measures. Ten of 14 centers (71.4%) completed at least one of these three measures for physical function while only two sites completed all three measures. The range of completion was 93%–98% for overall well-being and pain intensity scores and 69%–94% for physical function for the top four performing centers reporting data.

## Case studies

Four physician leaders in PR-COIN who previously reported successful collection of PROs were interviewed and shared their operational processes and lessons learned.

### PRO collection

Each of the high-performing sites initially started collection of PROs on paper. Prior to joining the LHN, PROs were collected primarily for the purposes of research registries or clinical trials, rather than for clinical care of the patient. Two of these sites had a strong culture of collecting PROs and reliable processes of paper-based data collection preceding joining PR-COIN. One site linked the collection of “Review of Systems” items to collection of pain score and overall wellbeing which may have contributed to the high collection rate. One site noted that a prior workflow utilized REDCap as the only access to PROs, which hindered physician engagement with the data. Physician interaction and access to data subsequently improved after the incorporation of an automated form in the EMR, which eliminated the need to log into a different system.

### Barriers

Time limitations were often cited as a barrier to collecting, reviewing, and acting on PROs results at point-of-care. A perceived or actual increased workload is a major barrier to collection and utilization of PROs by the clinical team.

All sites reported that utilizing the clinical Juvenile Arthritis Disease Activity Score (cJADAS) (13) in “treat to target” (5) discussions was the most common scenario where PROs are used by their colleagues. However, not all physicians at each site necessarily discussed answers of the PROs with patients, primarily citing time constraints as a barrier. Physicians were also concerned that additional time would be needed to better understand any discrepancies between the patient global assessment of overall wellbeing and the physician global assessment of disease activity. In general, this has not discouraged efforts for PRO collection across the rheumatology teams and, as a group, physicians recognized the importance of collecting patient perspectives.

While increased workload for physicians was cited as a common barrier to administration of PROs, use of automated systems to offset the workload was identified as a critical facilitator for PRO collection by each of these sites.

Other barriers to collection of specific PROMs include obtaining permission/license to use certain surveys (Table 2).

**Table 2:** Self-identified facilitators of PROs collection rates in PR-COIN.

Facilitators to PRO collection
PRO integration to EMR and learning network registry
Minimal burden (e.g., time and effort) for physician
Patient engagement in selection of measures
Presence of a clinical champion and project manager to encourage adoption by clinical team
Physician review of and use of PRO responses with patients to track patient's health status
Departmental leadership support and resources
Previous experience collecting PROs
Fostering a culture of PRO collection as standard practice
Adequate staffing to assist with collecting and documenting PROs
Adequate training for staff and clinicians
Presence of discrete response options
Automated reminders to both patients and staff members to complete/collect PROs
Barriers to PRO collection
Limited time
Lack of staff and resources administer PROs and enter data
Lack of resources to build IT infrastructure and oversee data transfer
Additional workload which interrupts clinical workflow
Lack of buy-in from individuals with interests (physicians, institutional leadership)
Low priority for institution
Lack of understanding of importance of PROs from patients/proxies
Lack of or suboptimal automation of PRO collection
Lack of adequate training of staff in data collection and data validation
Concern by physicians about alignment of PROs scores with physician assessment
Lack of availability of PROMs in different languages
Lack of interface to share and discuss PROs with patients
Difficulty standardizing PRO responses

PRO, patient reported outcomes.

### Facilitators

Two of the most effective facilitators of successful collection of PROs identified among all four sites were the roles of an engaged leadership team and information systems team for initial implementation and ongoing maintenance. An established culture of collecting PROs was also cited as an important facilitator (Table 2).

Leadership engagement was identified as a strong facilitator at all the sites, with three sites citing specific examples. A unique facilitator, conceived by the quality improvement physician champion and the section chief, employed at one site is a physician incentive, linked to the documentation of physician global score and joint count in a prespecified proportion of all patient encounters. While these are not PROs, the two measures coupled with a measure of patient overall well-being comprise the cJADAS (13). Providing maintenance of certification is another motivator for physician participation at this site.

Automated collection and calculation of PRO scores were consistently identified as a facilitator of successful PRO collection and clinician engagement. One site leader said, “the biggest thing I would say is to get the questionnaires electronic.” Another leader agreed, stating because PRO collection is “fully automated on the tablet, the burden for the nurses is really minimal.” One center described that the automated calculation of a composite disease activity measure, cJADAS (13), which incorporates a PRO (“overall well-being”), as a motivation for the clinical team to ensure that patients complete the PROM. The reason providers were invested in the PROM completion is because the cJADAS is the basis of a “treat to target” intervention used by providers to improve patient outcomes (5) and part of the critical data set of PR-COIN. Three of four sites indicated that the cJADAS is automatically calculated by the EMR. Electronic data transfer to the PR-COIN registry platform (4), is automated at three of the four sites interviewed. The fourth site currently relies on the nursing staff to screen for eligible patients prior to the office visit and to ask PRO questions during the rooming process. Scores are manually calculated by the physicians. This site plans to implement an automatic calculation model in the future. Successful transfer of data to the registry at this site was attributed to having a dedicated staff member who manually uploaded data. Thus, the involvement of an information systems team to build direct electronic transfer and provide tablets was identified as an important facilitator that would remove the burden of PRO collection from the clinical and research staff.

Physician engagement was another important facilitator. One site expressed the importance of having a project manager and physician champion with experience with quality improvement methodology, identification of clinically meaningful measures and practical aspects of implementation, such as frequency of releasing surveys.

Buy-in from other clinical staff was also cited as important. At one site, nursing staff were integral to the PRO collection process. There was a high level of engagement from nursing leadership (where they had the same nurse leader for the past 10 years).

Patient engagement is critical to the successful collection of PROs. One site specifically involved patients in designing the surveys, with careful attention to minimize burden and include PROs that patients felt were important. All sites noted that physician acknowledgment and utilization of PROs motivated patients to complete surveys. It provides “positive reinforcement that we are listening to them.” Other sites agreed, citing culture where “patients are just used to filling these out”, generally resulted in high fidelity, as did patient portal access and short length of surveys. One site reported conducting a study on the utilization of open note access, where patients were encouraged to review their own office notes. Patients reported that their efforts were validated when they saw their responses incorporated into their physician's notes.

### Lessons learned

Other important components of successful implementation included: practicing patience, making small, incremental changes, and establishing a unified workflow with the entire clinical team prior to implementation in order to maximize engagement.

To facilitate physician interaction with PROs at point of care, data were made available through multiple methods. Each site had a distinct tab in their EMR where PROs can be viewed. They also had a note template, which “pulled in” patient answers, making them immediately visible to the treating physician. Several sites also presented data in a flowsheet or dashboard, allowing results to be tracked over time, facilitating discussions with patients during their visit. One site programmed PROs to be released at regular intervals, with the option of setting up best practice alerts at pre-specified intervals (e.g., three months after treatment change) to review “treat to target” goals.

Training on the workflow for collecting PROs and their use in direct patient care, as well as the introduction to quality improvement efforts were provided to all new trainees and faculty at each of these sites.

Regular meetings led by the physician champion with the clinical team to review data and disease activity scores were deemed important at reinforcing the collection of PROs and maintain high rates of completion. Sharing the impact of improvement efforts on a regular basis “swayed even the skeptics in the group.” At one site, graphs of anonymized PRO completion rates for the division were published monthly and higher-performing providers were invited to share their best practices with the team. Additionally, the physician champion had conducted annual one-on-one meetings with each clinician to review individual's and center performance (completion rates and overall disease scores/remission) and provided support to improve rates.

Participation in PR-COIN was credited for providing structure, motivation, and justification for existing PRO collection workflows. At one site, the formalized collection of PROs was established after joining PR-COIN. The physician leader at this site noted that the contributing data to a registry validates the value of effort from the physicians, nurses and data team.

In addition to technical support and guidance from PR-COIN, these leaders proposed the creation of a formal guide to improve PRO collection, establishing high-level steps and milestones, performance objectives of division chief, quality improvement physician champion, information systems teams, parent engagement, and ancillary and research staff. Furthermore, formal recommendations from an LHN can serve as a powerful advocate to persuade local hospital leadership of the significance and impact of PRO collection.

All four sites envisioned the creation of a patient-facing platform in the future, which would enable patients to view their PROs over time. Three sites also planned to have PROs available in other languages in order to improve delivery of care and communication with non-English speaking patients.

## Discussion

As healthcare increasingly focuses on patient-centered care, it is imperative that healthcare providers, researchers, and policy makers collectively support and adopt processes to enable reliable and complete collection of PROs. Reaching a consensus on a core set of PROs that accurately reflect patients' needs and desires, while minimizing the burden on patients and proxy reporters, is a primary step to achieving this goal. This would serve as a foundation for standardizing processes and systems to optimize the collection and use of PROs to improve health outcomes.

Through a consensus-based approach with patients, parents/caregivers and healthcare providers, the Outcome Measures in Rheumatology (OMERACT) Juvenile Idiopathic Arthritis (JIA) Workgroup has created a detailed definition and description for the two target domains in the patient perception of overall well-being related to disease (6, 14). Through the PR-COIN Parent Workgroup, patient and parent voices have informed the PROs collection (3).

Our survey revealed varying PRO collection rates and process across the LHN. The self-reported nature of the survey has limitations as it may not accurately reflect the completion of PRO data fields or reliable transfer into the shared LHN registry. Integration of PROs into EMRs was identified as a facilitator to PRO collection from both the survey and interviews we conducted. EMR integration requires an upfront investment but will enable healthcare providers to efficiently collect data longitudinally. As an LHN, building a system that digitally collects PROs, having alignment of PROs across the network and the collection of responses using standardized scales would facilitate the network's ability to compare outcomes of treatment across network sites using PROs.

To increase the incorporation of patients’ perspective in their clinical care, an equally critical consideration is accessibility of PROs in the EMR for both clinicians and patients. Involving clinicians in the design of PRO displays may improve their ability to act on the information while minimizing burden of additional “clicks”. Our interviews revealed that when patients see their data being utilized in treatment decisions, it helps them understand the rationale for completing and motivates them to complete PROs. Accessibility of PROs for clinicians and patients highlights the importance of technical support to build and maintain an accessible interface.

In addition to the importance of integrating PRO data into the EMR and ensuring accessibility of PROs for both clinicians and patients, the survey and interviews revealed other facilitators for successful PRO collection: minimizing clinician time and effort (i.e., in administration/calculation), having a designated physician champion and project manager, providing feedback on collection rates to the clinical team, and fostering a culture that values PRO collection within the department or institution. Notably, each of the four PR-COIN sites interviewed had an established paper-based PRO collection process before integrating it into the EMR. This experience likely assisted in establishing the feasibility of PRO collection and utilization prior to hardwiring this process electronically. While there is no single effective model, considerations for planning and implementation are central to successful and sustainable PRO collection.

Lessons learned from other successful organization-wide implementation of PRO collection and utilization (15, 16) echo those learned from our LHN. Key factors include physician and administration engagement, presence of a clinical champion, prior experience with PRO collection, and payer incentive contracts.

Based on these insights, PR-COIN is now well-positioned to develop a toolkit (17). The toolkit will outline sample workflows, implementation strategies and other resources for collecting PROs, similar to the approach which has been used in a learning health network for rheumatoid arthritis patients (18).

For a LHN focused on patient outcomes, it is essential to administer and complete PROs with high reliability to measure performance and guide improvement in areas prioritized by patients. Currently, there is limited guidance on standardization of PRO collection within a LHN, resulting in variable rates of completion. Through understanding our current ability to systematically collect PROs across all PR-COIN sites and exploring successful implementation of PRO collection both within and outside our network, we share lessons learned and identify key factors that contribute to the successful spread of this important practice.

## Ethical considerations

The PR-COIN registry was approved by Seattle Children's Institutional Review Board (IRB), which serves as the IRB of record for Seattle Children's Hospital for following relying participating sites: Stanford University, University of Mississippi, Children's Wisconsin, Northwell Health/Cohen Children's Medical Center, Baylor College of Medicine/Texas Children's Hospital, University of Minnesota, Phoenix Children's Hospital, Nationwide Children's Hospital, Medical University of South Carolina, Hospital for Special Surgery, Hackensack Meridian Health, Cincinnati Children's Hospital Medical Center, Children's Mercy Kansas City, Children's Hospital of Philadelphia, Boston Children's Hospital, and University of Alabama at Birmingham. Due to institutional regulatory policies and local or provincial laws and regulations. The registry was approved by a local IRB for 6 participating sites: Levine Children's/Atrium Health (Charlotte, NC, United States), London Health Sciences Centre/Lawson Health Research Institute (London, ON, Canada), McMaster University (Hamilton, ON, Canada), Nemours Orlando (Orlando, FL, United States), Penn State Children's Hospital (Hershey, PA, United States), and The Hospital for Sick Children/SickKids (Toronto, ON, Canada).

PR-COIN uses a collaborative learning health system approach to improve quality of care and outcomes for children with JIA. PR-COIN currently has 23 participating sites from academic pediatric medical centers throughout the United States and Canada. PR-COIN is led by a coordinating center which provides quality improvement consultation, quality improvement education, maintenance of certification opportunities, data management, data analytics, legal and regulatory supervision, project development and oversight, and overall support to the network.

## Data Availability

The raw data supporting the conclusions of this article will be made available by the authors, without undue reservation.
