# Supplementary material for: Achieving reliable patient reported outcomes collection to measure health care improvement in a learning health network: lessons from pediatric rheumatology care and outcomes improvement network
Source: Front Pediatr. 2025 Jan 8;12:1443426. doi: 10.3389/fped.2024.1443426 (PMC11753412; doi:10.3389/fped.2024.1443426)
Supplement: Supplementary file 1 [file Datasheet1.docx]

# Supplement 1. Survey Questions

**INSTRUMENTS**

Do you collect patient-reported data/outcomes as part of clinical care at your center?

- Yes
- No

Which patient-reported data/outcomes do you currently collect as part of clinical care at your center?

- Child Health Assessment Questionnaire (CHAQ)/Health Assessment Questionnaire (HAQ
- PROMIS-Anxiety
- PROMIS-Depressive symptoms
- PROMIS-Fatigue
- PROMIS-Mobility
- PROMIS-Upper extremity
- PROMIS-Pain interference
- Juvenile Arthritis Quality of Life Questionnaire (JAQQ)
- Quality of My Life (QoML)
- Patient Health Questionnaire-2 (PHQ-2)
- Patient Health Questionnaire-9 (PHQ-9)
- Juvenile Arthritis Functional Assessment Report (JAFAR)
- Juvenile Arthritis Functionality Scale (JAFS)
- Juvenile Arthritis Functional Status Index (JASI)
- PedsQL RHE Child
- PedsQL RHE Parent
- PedsQL Core Child
- PedsQL Core Parent
- Outcome Measure Child Health Questionnaire (CHQ)
- Methotrexate Intolerance Severity Score (MISS)
- Bath Ankylosing Spondylitis Disease Activity Index (BASDAI)
- Patient global assessment
- Patient global-overall well-being
- Patient global-disease activity
- Pain-intensity score (e.g., 0-10)
- Transition readiness
- Juvenile Arthritis Multidimensional Assessment Report (JAMAR)
- Pain Symptom Assessment Tool (PSAT)
- Morning stiffness
- Review of systems
- EQ-5D
- Other _________________________________________

Are there any hospital-mandated PROs (e.g., suicide screening, PHQ9)?

- Yes
- No

If so, which ones?

________________________________________________________

Do you have any plans to add/remove instruments?

- Yes
- No

If so, which ones?

________________________________________________________

Add: ____________________________________________________

Remove: _________________________________________________

What scale do you use to collect pain-intensity related PROs?

Visual Analogue Scale (Line)

Numeric Rating Scale

Wong-Baker Faces Scale

Other _____________________________________________________

What is the is range of pain-intensity related PROs?

- 0-100
- 0-10

What increments do you use to measure pain-intensity related PROs?

- 1 unit increments
- 0.5 unit increments

What scale do you use to collect physician global assessment of disease activity?

- Visual Analogue Scale (Line)
- Numeric Rating Scale

What is the range?

- 0-100
- 0-10

What increments do you use to measure physician global score?

- 1 unit increments
- 0.5 unit increments

**ADMINISTRATION PROCESS**

What personnel are involved in administering of PROs?

- Patient/caregiver (self-entered)
- Front desk staff
- Research coordinator
- Nurses/Medical assistant
- Physician
- Volunteer
- Other ________________________________________________

Which EMR does your center use?

- Epic
- Cerner
- AllScripts
- Other _________________________________
- Don't use EMR

What is the mode of collection at your center?

- Paper
- Electronic

How are your PROs electronically captured?

- iPad/tablet in clinic
- Patient Portal
- REDCap
- Website
- Smartform
- Other _____________________________________________________

On average, how long does it take staff to ask PROs? (enter 0 if patient/parent-entered)

___________________________________minutes

On average, how much time does it take for patients/parents to complete PROs on their own?

___________________________________minutes

How do your PRO scores get calculated?

- Automated
- Manual

Who calculates the score?

- Attending physician
- Practitioner (NP/PA/PT/OT)
- Nurse
- Fellows/Trainees
- Medical assistant
- Research personnel
- Volunteer
- Other __________________________________________

Where are the scores calculated?

- Electronic medical record (flowsheet)
- Electronic medical record (SmartForm)
- REDCap system
- Written in note (therefore not calculated)
- Other __________________________________________

Are PROs automatically uploaded to EMR via tablet/REDCap after patient/parent completes it? i.e., no human intervention

- Yes
- No
- Some

Who enters the PROs into the EMR?

- Attending physician
- Practitioner (NP/PA/PT/OT)
- Nurse
- Fellows/Trainees
- Medical assistant
- Research personnel
- Volunteer
- Other __________________________________________

On average, how long does it take staff to enter PROs into EMR or database per patient? (enter 0 if patient/parent-entered)

________________________________________ minutes

Please elaborate as needed _____________________________________________

Are the PROs stored as discrete data elements in medical records so that they can be automatically extracted?

- Yes
- No

Do you have any plans to alter the mode of data collection (i.e., from paper to tablet)?

- Yes
- No

How do you plan to alter the mode of PRO collection?

____________________________________________________________

**STATISTICS**

Who is asked to complete PROs?

Are your patients over a certain age instructed to answer PROs without their caregiver?

- Yes
- No

What age do you make this request?

______________________________________________________________

Please share the instructions

_____________________________________________________________

Approximately what proportion of JIA patient visits at your institution are PROs?

- 0
- 1-25%
- 26-50%
- 51-75%
- 76-99%
- 100%

Approximately what proportion of JIA patient visits at your institution are PROs completed?

- 0
- 1-25%
- 26-50%
- 51-75%
- 76-99%
- 100%

When do you request patients to complete PROs?

- Before the visit
- During the visit
- After the visit

Are completed PROs available at the time of the patient's visit?

- Yes (100%)
- No (0)
- Some

Approximately what proportion of completed PROs are available during the patient's visit?

- 0
- 1-25%
- 26-50%
- 51-75%
- 76-99%
- 100%

Which PROs are collected for only clinical purposes, only research, both clinical and research purposes?

|  | Clinical Only | Research Only | Clinical and Research | Do Not Collect |
| --- | --- | --- | --- | --- |
| CHAQ |  |  |  |  |
| PROMIS-Anxiety |  |  |  |  |
| PROMIS-Depression |  |  |  |  |
| PROMIS-Fatigue |  |  |  |  |
| PROMIS-Mobility |  |  |  |  |
| PROMIS-Upper extremity |  |  |  |  |
| PROMIS-Pain interference |  |  |  |  |
| JAQQ |  |  |  |  |
| QoML |  |  |  |  |
| PHQ-2 |  |  |  |  |
| PHQ-9 |  |  |  |  |
| JAFAR |  |  |  |  |
| JAFS |  |  |  |  |
| JASI |  |  |  |  |
| PedsQL RHE Child |  |  |  |  |
| PedsQL RHE Parent |  |  |  |  |
| PedsQL Core Child |  |  |  |  |
| PedsQL Core Parent |  |  |  |  |
| CHQ |  |  |  |  |
| MISS |  |  |  |  |
| BASDAI |  |  |  |  |
| Patient global assessment |  |  |  |  |
| Patient global-overall well-being |  |  |  |  |
| Patient global-disease activity |  |  |  |  |
| Pain intensity score (e.g., 0-10) |  |  |  |  |
| Transition readiness |  |  |  |  |
| JAMAR |  |  |  |  |
| PSAT |  |  |  |  |
| Morning stiffness |  |  |  |  |
| Review of systems |  |  |  |  |
| EQ-5D |  |  |  |  |
| Other _________________ |  |  |  |  |

**OPINIONS**

What are facilitators that help in the collection of PROs?

- Preprinted forms
- Patient portal
- REDCap
- SmartForm
- Trained personnel
- Adequate number of personnel
- Supportive personnel
- Institutional structure
- Institutional priority/resources e.g., providing tablets
- Other _______________________________________________

What barriers limit you from administering PROs at each visit?

- Provider has no time to review scores
- Limited staff to enter data into EMR
- Providers are not interested in collecting this data in their patients
- Providers have limited knowledge on how to act on the responses of the PROs
- Patients have no time
- Patients have no interest
- Language
- Not an institutional priority/resources
- Other _______________________________________________

In your opinion, which are the top 3 most important PROs to collect?

|  | 1 | 2 | 3 |
| --- | --- | --- | --- |
| CHAQ |  |  |  |
| PROMIS-Anxiety |  |  |  |
| PROMIS-Depression |  |  |  |
| PROMIS-Fatigue |  |  |  |
| PROMIS-Mobility |  |  |  |
| PROMIS-Pain Interference |  |  |  |
| JAQQ |  |  |  |
| QoML |  |  |  |
| PHQ-2 |  |  |  |
| PHQ-9 |  |  |  |
| JAFAR |  |  |  |
| JAFS |  |  |  |
| JASI |  |  |  |
| PedsQL RHE Child |  |  |  |
| PedsQL RHE Parent |  |  |  |
| PedsQL Core Child |  |  |  |
| PedsQL Core Parent |  |  |  |
| CHQ |  |  |  |
| MISS |  |  |  |
| BASDAI |  |  |  |
| Patient global assessment |  |  |  |
| Patient global-overall well being |  |  |  |
| Patient global-disease activity |  |  |  |
| Pain intensity score (e.g., 0-10) |  |  |  |
| Transition readiness |  |  |  |
| JAMAR |  |  |  |
| PSAT |  |  |  |
| Morning stiffness |  |  |  |
| Review of systems |  |  |  |
| EQ-5D |  |  |  |
| Other ______________________ |  |  |  |

Please share why you think these are the 3 most important PROs

_______________________________________________________________________

Please share additional information about PROs that you feel is important

_______________________________________________________________________

**SITE DEMOGRAPHICS**

Number of faculty

_______________________________________________________________________

Number of fellows

_______________________________________________________________________

Number of allied health members (e.g., nurse, medical assistant)

_______________________________________________________________________

Are you an urban or rural site?

- Urban
- Rural

Which site do you represent? (optional)

- Boston Children's Hospital
- Children's Hospital of Philadelphia
- Children's Mercy Kansas City
- Children's of Alabama
- Cincinnati Children's Hospital Medical Center
- Cleveland Clinic
- Cohen Children's Medical Center of New York
- Hackensack University Medical Center
- Hospital for Special Surgery
- Levine Children's Hospital
- McMaster Children's Hospital
- Medical College of Wisconsin
- Medical University of South Carolina
- Nationwide Children's Hospital
- Nemours
- Penn State Health Children's
- Phoenix Children's Hospital
- Seattle Children's Hospital
- Stanford Children's Health
- Texas Children's Hospital
- The Hospital for Sick Children
- University of Minnesota
- University of Mississippi
