# Supplementary material for: Achieving reliable patient reported outcomes collection to measure health care improvement in a learning health network: lessons from pediatric rheumatology care and outcomes improvement network
Source: Front Pediatr. 2025 Jan 8;12:1443426. doi: 10.3389/fped.2024.1443426 (PMC11753412; doi:10.3389/fped.2024.1443426)
Supplement: Supplementary file 2 [file Datasheet2.docx]

Interview guide

- What is the importance of physician engagement in PRO collection? What, if any, have been barriers to physician engagement? What interventions help to make integration seamless/minimize additional demands on physician time and effort?
- What role does clinic administrator/hospital leadership play in successful PRO collection? how did you increase engagement of administrators and leadership?
- What is your view of patient engagement for successful PRO collection, including challenges and solutions
- What is your PRO collection process?
- What are the most important facilitators of PRO collection at your site?
- What are lessons learned from implementation of PRO collection at your site?
