# Supplementary material for: Achieving reliable patient reported outcomes collection to measure health care improvement in a learning health network: lessons from pediatric rheumatology care and outcomes improvement network
Source: Front Pediatr. 2025 Jan 8;12:1443426. doi: 10.3389/fped.2024.1443426 (PMC11753412; doi:10.3389/fped.2024.1443426)
Supplement: Supplementary file 4 [file Datasheet4.docx]

Supplement to COREQ Checklist Items

The interviewer (NP) conducted 1:1 interviews with each of the physician site leaders, who were known to her prior to the interview as colleagues and co-investigators in the PR-COIN Learning Health Network. NP holds an MD and currently practices as a pediatric rheumatologist. We have no reason to believe that any of the researchers’ personal experiences or life histories would have influenced their approaches to data collection and analysis in a noteworthy way.

Physician leaders of 4 sites who had previously shared successful collection of PROs were invited to share their experiences. All four physicians agreed to participate and none dropped out.
